# Lumbar Intraspinal Osteolipoma Presenting as Cauda Equina Syndrome: A Case Report and Review of Literature

**DOI:** 10.1155/2018/1945149

**Published:** 2018-10-22

**Authors:** S. Dilip Chand Raja, Rishi Mugesh Kanna, Ajoy Prasad Shetty, S. Rajasekaran

**Affiliations:** Department of Spine Surgery, 313 Mettupalayam Road, Ganga Hospital, Coimbatore, India

## Abstract

**Introduction:**

Osteolipomas are uncommon variants of lipoma. These lesions have been usually reported to arise from the oral cavity, brain, and neck and scarcely from the knee and thigh. Intraspinal osteolipomas are rare. A single case of intraspinal osteolipoma has been reported in the cervical and thoracic spine in the literature. To our knowledge, there is no report of osteolipomas in the lumbar spine.

**Case Presentation:**

We report a very rare case of a solitary lumbar intraspinal osteolipoma, presenting as a cauda equina syndrome. The intraspinal osteolipoma was excised en bloc and fusion was performed as it required partial resection of the facet joint within 24 hours of presentation. He has since then improved neurologically, and there has been no recurrence of the lesion so far. The clinical presentation, radiological characteristics, treatment course, and histopathological features of this lesion along with the clinical outcomes and a pertinent literature review were done and have been discussed.

**Discussion:**

The heterogeneous signal intensity of the lesion in MRI differentiates it from other dural-based lesions, and this should raise suspicion of an osteolipoma, which warrants a CT. Although intraspinal osteolipomas are benign lesions and generally have good prognosis, they need to be removed en bloc as they may result in rapid neurological deterioration.

## 1. Introduction

Lipomas are the most common benign mesenchymal tumors [1]. Osteolipomas are rare variants and constitute only 1% of all lipomas and are alternatively referred to as ossifying lipomas or osseous lipomas [2]. Most of the reports in literature have reported their existence in oral cavities, head, brain, knee, and thigh [3–6]. However, there are only countable cases of osteolipoma related to the spine. There are only three reports of solitary intraspinal osteolipomas until now—two in the cervical region and one in the thoracic spine. We present the first case of lumbar intraspinal osteolipoma and have discussed its clinical presentation, diagnostic challenges, and management along with a pertinent review of literature.

## 2. Case Report

A 36-year-old male presented to the outpatient department with a history of low backache for the past one year, associated with typical claudication symptoms, left-sided unilateral numbness, and paresthesia of the foot. He gave no history of radicular pain, but he complained of progressive reduction in claudication distance. He had experienced a sudden deterioration in gait along with urinary incontinence, following a trivial traumatic fall, one week before presentation. There was no history of constitutional features. On examination, he had a bilateral high-stepping gait due to foot drop. Symmetrical weakness of both the L4 and L5 roots (MRC grade 0/5) and partial weakness of the S1 root (MRC grade 3/5) was noted with nondermatomal sensory disturbances. Deep tendon reflexes of the lower limbs were absent bilaterally. Though anal tone was normal, saddle anesthesia was present. Postvoidal ultrasonogram of the urinary bladder revealed a residual urine volume of 250 ml, thus confirming a neurogenic bladder.

**Figure 1:**
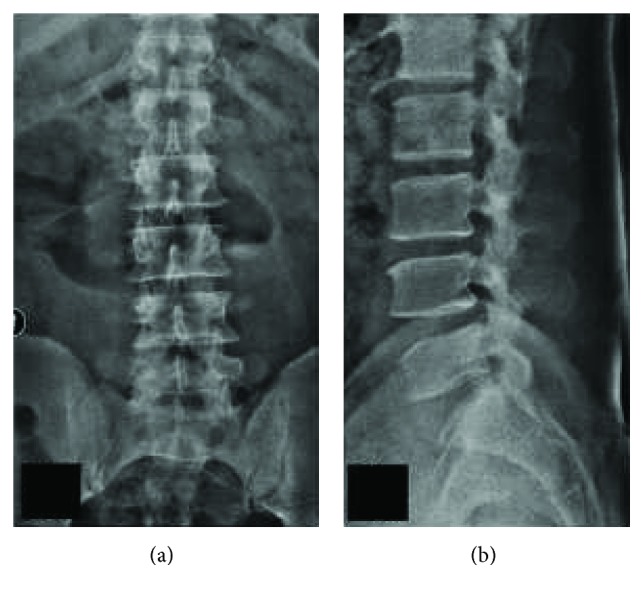
(a) and (b) Minimal spondylotic changes seen in plain radiography of the spine with no features of gross instability.

**Figure 2:**
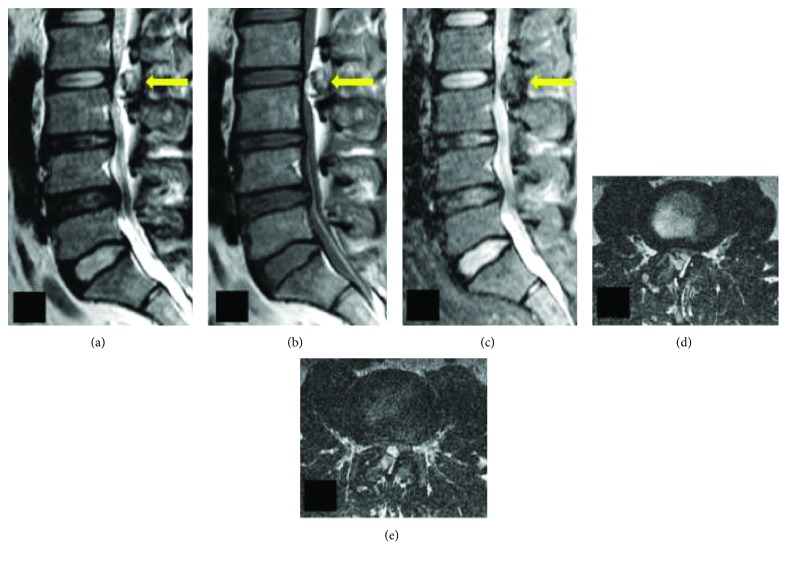
(a and b) T2- and T1-weighted sagittal MRI images showing a large epidural intraspinal posterior epidural lesion with hyperintense signals in the periphery and hypointense signals at the center at the L2-L3 level with severe thinning of the thecal sac. The hyperintense region becomes hypointense in the fat-suppressed sequence (c) confirming the presence of fat in the periphery and bone tissue in the center of the lesion. (d and e) Axial images demonstrating adjacent epidural fatty hypertrophy contributing to stenosis in addition to the lesion reducing the caliber of dura to near nonexistence.

**Figure 3:**
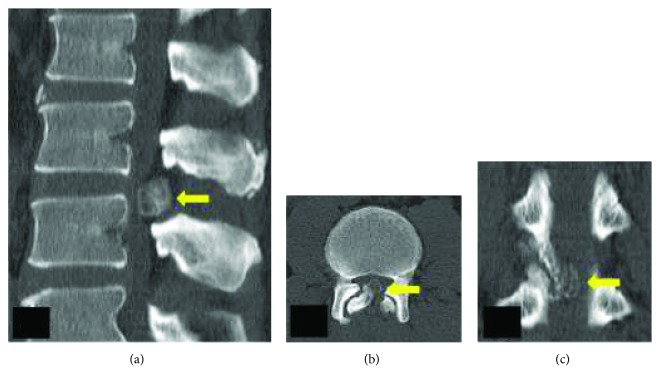
(a) Sagittal CT image shows the huge osseous lesion causing severe compromise of the spinal canal. (b and c) Axial and coronal images demonstrating the attachment to the right L2 inferior articular process.

**Figure 4:**
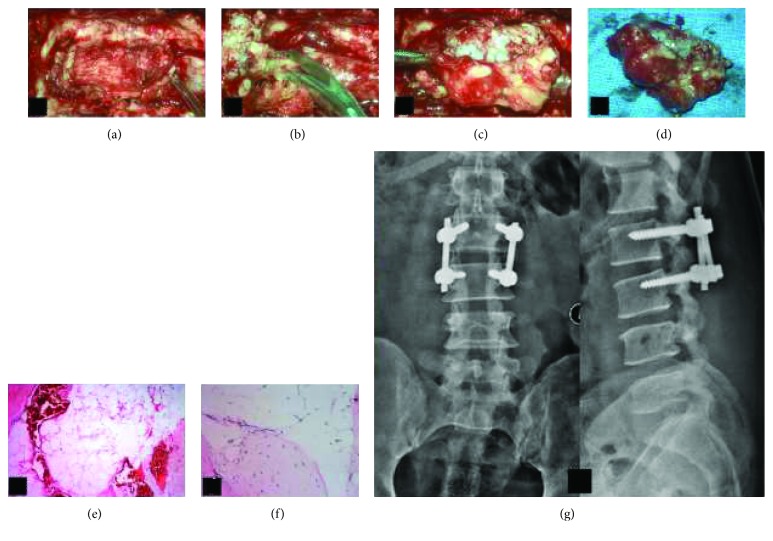
(a) Intraoperative microscopic view image—bone window created around the lesion. (b) Adherent soft tissue being removed from beneath the lesion. (c) En bloc removal of the lesion. (d) Gross appearance of the lesion appearing grey white and measuring 2 cm × 1.5 cm × 1.5 cm. (e) Histopathological image showing mature well-capsulated adipose tissue with intermittent osteoid. (f) Lamellar osseous tissue adjacent to adipose tissue visible on higher magnification. (g) Plain radiography following complete excision of tumor and fusion at the L2-L3 level.

Plain radiography of the lumbar spine did not show any gross feature of instability and was inconclusive (Figure 1). Magnetic resonance imaging (MRI) of the lumbar spine revealed a solitary intraspinal posterior epidural lesion of 1.8 × 1.5 × 0.5 cm at the L2-L3 level with heterogeneous signal intensities and adjacent epidural fatty hypertrophy contributing to severe canal stenosis (3 mm). The cauda equina was severely compressed and was almost not visible (Figure 2). Owing to the heterogeneous signal intensities, computerized topography (CT) was performed which revealed the presence of an osseous lesion attached to the right L2 inferior articular process causing severe secondary canal stenosis (Figure 3). Considering the recent-onset neurological deficit, the patient was advised surgical decompression and excision biopsy at the earliest. The patient was positioned prone on a Relton Hall frame under general anesthesia. A standard midline posterior approach was employed and L2 and L3 lamina were exposed. The spinous process was removed and using a motorized burr, a rectangular trough was created surrounding the lesion. The lamina was thinned out using a burr to avoid further insult to the dural sac, and then using a Kerrison ronguer, laminectomy was completed all around the lesion under microscopic guidance (Figure 4). A small osteotome was used to remove the attachment on the right side which required partial removal of the facet joint. The lesion was then held, and the adherent soft tissues beneath the lesion were removed, resulting in en bloc removal and complete decompression of the cauda equina. Fusion was performed, as the procedure involved partial facet joint resection. The lesion was sent for histopathological examination. There were no adverse events or postoperative complications. The patient was mobilized with bilateral orthoses, and bladder training was initiated.

At 4 weeks, there was an improvement in his urinary symptoms, and by 12 weeks partial neurological recovery (MRC grade 3/5 motor power) in bilateral L4 and L5 was observed. The S1 root power increased by one grade (MRC grade 4/5). His gait improved thereafter, and he was able to return to his normal activities by 6 months with further neurological improvement by 1 grade in all roots.

The gross specimen measured 2 cm × 1.5 cm × 1.5 cm. It was greyish white in color and firm to hard in consistency (Figure 4(d)). It had a well-defined capsule and had the feel of particulate materials on cut sections. Histopathological examination revealed the presence of bone, cartilage and ligamentous tissue, and zones abutting all these composed of mature adult-type encapsulated adipose tissue (Figures 4(e) and 4(f)). This confirmed the diagnosis of benign osteolipoma.

## 3. Discussion

Lipomas are ubiquitous and are the most common mesenchymal tumors comprising mature adipose tissue. Occasionally, lipomas may contain other types of mesenchymal tissue and such variants are named accordingly as fibrolipoma (connective tissue), angiolipoma (blood vessels), chondrolipoma (cartilage), and osteolipoma (bone). Osteolipomas are extremely rare variants and constitute only 1% of all lipomas; they are alternatively referred to as ossifying lipomas or osseous lipomas [7].

Most of the reports in literature reveal their existence in oral cavities, head, brain, knee, and thigh, and there are only countable reports of osteolipoma related to the spine [3–9]. There are only three reports of solitary intraspinal osteolipomas, two in the cervical region and one in the thoracic spine. In 2001, Lin et al. was the first to report on intraspinal osteolipoma. The tumor was located in the cervical spine and was diagnosed wrongfully as a case of intradural extramedullary tumor radiologically [10]. In 2011, Yang et al. first reported the successful treatment of the largest thoracic intraspinal osteolipoma [11]. Later in 2016, the second case of cervical intraspinal osteolipoma presenting as isolated dorsal column dysfunction was reported by Aiyer et al. [12]. Though lipomas are common in the lumbar region, an osteolipoma has been reported only once. In 2005, Jaiswal et al. reported a case of a lumbar spinal ossifying lipoma in the subcutaneous region in communication with an intradural lipoma associated with spinal dysraphism [13]. However, there was no intraspinal osseous component, thus making our case to be the first report on lumbar intraspinal osteolipoma. Various hypothesis have been postulated regarding the pathology of osteolipomas including repeated microtrauma, metaplasia of fibroblasts to osteoblasts, and chondroossification of preexisting mesenchymal cells. Translocations involving the 12q13-15 chromosome have been observed in osteolipomas [14, 15].

## 4. Diagnosis of Osteolipomas

Plain radiographs fail to reveal the lesion. MRI would demonstrate a mass occupying the spinal canal. A clarity of thought is essential in evaluating these patients, to differentiate it from the more common dural-based tumors. Heterogeneous signals within the lesion should raise the suspicion of the presence of both fat and bone in the lesion [16]. Calcifying intradural tumors present in a similar manner. CT imaging is essential, as it clinches the diagnosis of osteolipoma and helps in delineating its margins. Surgical modality is the treatment of choice, and early diagnosis and decompression of such lesions can avoid permanent neurological deficits. CT helps in determining the extent of the lesion, reveals any attachments, and aids in planning surgical decompression. It is vital to get to the interface between the dura and lesion to avoid further neurological worsening, secondary to the handling of neural tissues. However, due to severe epidural congestion it might not always be possible. In such scenarios, it is better to remove the lesion from outside in by thinning out the lamina where dural compression is minimal and then do a wide decompression to facilitate en bloc removal of the lesion after freeing it from its surroundings. Generally, the prognosis of benign osteolipoma is fairly good as observed in our case. We have followed him up for 2 years since the surgical intervention and there has been no recurrence or complications.

## 5. Conclusion

This case represents the first report of an intraspinal osteolipoma of the lumbar spine presenting as a cauda equina syndrome. The characteristic signal intensity variations in different MRI sequences and the presence of osseous tissue in CT provide excellent aid in diagnosis. The histopathological presence of osteoid and mature adipose tissue confirms the diagnosis of benign osteolipomas and are best managed surgically by complete resection of the mass and usually carry good prognosis.

## References

[1] Weiss S. W. (1996). Lipomatous tumors. *Monographs in Pathology*.

[2] Obermann E. C., Bele S., Brawanski A., Knuechel R., Hofstaedter F. (1999). Ossifying lipoma. *Virchows Archiv*.

[3] Castilho R. M., Squarize C. H., Nunes F. D., Pinto D. S. (2004). Osteolipoma: a rare lesion in the oral cavity. *British Journal of Oral and Maxillofacial Surgery*.

[4] Sinson G., Gennarelli T. A., Wells G. B. (1998). Suprasellar osteolipoma: case report. *Surgical Neurology*.

[5] Heffernan E. J., Lefaivre K., Munk P. L., Nielsen T. O., Masri B. A. (2008). Ossifying lipoma of the thigh. *The British Journal of Radiology*.

[6] Val-Bernal J. F., Val D., Garijo M. F., Vega A., González-Vela M. C. (2007). Subcutaneous ossifying lipoma: case report and review of the literature. *Journal of Cutaneous Pathology*.

[7] Saghafi S., Mellati E., Sohrabi M., Raahpeyma A., Salehinejad J., Zare-Mahmoodabadi R. (2008). Osteolipoma of the oral and pharyngeal region: report of a case and review of the literature. *Oral Surgery, Oral Medicine, Oral Pathology, Oral Radiology, and Endodontology*.

[8] Bohm K. C., Birman M. V., Silva S. R. (2011). Ossifying lipoma of C1-C2 in an adolescent. *Journal of Pediatric Orthopaedics*.

[9] Yang J. S., Kang S. H., Cho Y. J., Choi H. J. (2013). Pure intramuscular osteolipoma. *Journal of Korean Neurosurgical Society*.

[10] Lin Y. C., Huang C. C., Chen H. J. (2001). Intraspinal osteolipoma. case report. *Journal of Neurosurgery: Spine*.

[11] Yang J., Li S., Kang A., Chen X., Su B., Jin Y. (2012). A giant intrathoracic osteolipoma: a case report and review of the literature. *International Journal of Surgery Case Reports*.

[12] Aiyer S. N., Shetty A. P., Kanna R., Maheswaran A., Rajasekaran S. (2016). Isolated dorsal column dysfunction due to an intraspinal osteolipoma—case report and review of literature. *Journal of Clinical Orthopaedics and Trauma*.

[13] Jaiswal A. K., Garg A., Mahapatra A. K. (2005). Spinal ossifying lipoma. *Journal of Clinical Neuroscience*.

[14] Plaut G. S., Salm R., Truscott D. E. (1959). Three cases of ossifying lipoma. *The Journal of Pathology*.

[15] Katzer B. (1989). Histopathology of rare chondroosteoblastic metaplasia in benign lipomas. *Pathology - Research and Practice*.

[16] Fritchie K. J., Renner J. B., Rao K. W., Esther R. J. (2012). Osteolipoma: radiological, pathological, and cytogenetic analysis of three cases. *Skeletal Radiology*.

